# Estrogen and Androgen Hormone Levels Modulate the Expression of PIWI Interacting RNA in Prostate and Breast Cancer

**DOI:** 10.1371/journal.pone.0159044

**Published:** 2016-07-14

**Authors:** Çağrı Öner, Didem Turgut Coşan, Ertuğrul Çolak

**Affiliations:** 1 Eskişehir Osmangazi University, Medical Faculty, Department of Medical Biology, 26480, Eskişehir/Turkey; 2 Eskişehir Osmangazi University, Medical Faculty, Department of Biostatistics and Medical Informatics, 26480, Eskişehir/Turkey; National Institute of Technology, Rourkela, INDIA

## Abstract

PIWI interacting RNAs (piRNAs), a member of non-coding RNA, originate from intergenic repetitive regions of the genome. piRNA expressions increase in various cancers and it is thought that this increase could be caused by hormones. We aimed to determine the effects of hormones on piRNA expression in breast and prostate cancer. High viability and a decrease in adhesion were observed at the concentrations of the highest proliferation. Furthermore, an increase in adhesion was also observed in MDA-MB-231 cells. After hormone treatment, while *piR-651* expression had increased both breast and prostate cancer cell lines, *piR-823* expressions increased in prostate cancer cell lines and only in the breast cancer cell line which was malignant. Thus, it was determined that *piR-823* might show different expressions in different type of cancers.

## Introduction

Gender dependent steroid hormones play an important role in the development and mechanism of cancer of the reproductive system, particularly in prostate cancer in males and uterus and breast cancer in females [1,2]. Androgen, a steroid hormone, plays an important role in the development of prostate cancer [3]. Prostate cancer develops in two ways, being either androgen-dependent or androgen-independent. Androgen-dependent prostate cancer cells require, in the early stages of the development of prostate cancer, the 5α-dihydrotestosterone to be converted from testosterone by the 5α-reductase enzyme system. Androgen-independent prostate cancer cells, however, are seen in the advanced stages of cancer development and do not need androgen in order to grow after these stages. The inefficacy of androgen in these types of cancer cells is associated with the changes, such as mutation, amplification or deletion, in the androgen receptor [2,4,5]. Breast cancer, the most common type of cancer after lung cancer, originates from cells in the tissues producing or carrying human breast milk, 80% of which are the epithelial layers of the lactiferous ducts [6] which contain estrogen receptors, and approximately 50 to 85% of breast tumors contain estrogen receptors and are seen in the cytosol [7].

The importance of non-coding RNAs in the development and prognosis of most diseases, particularly in cancer, has been increasing more and more, and the studies which have been carried out mark their importance as epigenetic regulators [8,9]. piRNAs and PIWI proteins are still being studied in order to obtain knowledge about their role in pathogenic mechanisms, such as tumorigenesis [10,11]. piRNAs maintain genome integrity by epigenetically silencing the transposons through DNA methylation, especially in gamete stem cells. piRNAs were identified in male germline cells during DNA methylation-mediated transposon silencing by affecting the expression of *DNMT3*, which is a DNA methyltransferase [10,12–14]. It is known that they are highly expressed at a length of 26–31nt and in the testes. In mammals, piRNAs were first identified in 2006 in the cloning of small RNAs associated with PIWI proteins in the mammalian system [15–17]. piRNAs act as a sequence-specific guide for PIWI proteins, and the PIWI protein-piRNA pathway in gamete stem cells is thought to be important for DNA methylation-mediated transposon silencing [12,13,18,19]. Genetic studies in mice, Drosophila, and Zebrafish reported that piRNAs are expressed more than any other short non-coding RNAs during germinal development and in the testes [20–22]. Though piRNAs are mostly thought to be specific to germline cells, recent studies have shown that they also play important roles in non-gonadal cells [10,23–25], such as in long-term memory functions, DNA methylation, epigenetic regulations, the silencing of transposable and non-transposable elements, and the stability of primary cells composing the gamete cells [26].

Our study investigates the piRNAs in hormone-dependent and hormone-independent cancer cells, and its aim is to determine whether these piRNAs play effective roles only in hormone-dependent cancers or the same is true for hormone-independent cancers as well. For this purpose, we treated androgen-dependent and androgen-independent prostate cancer cells (LNCaP and PC-3) with an androgen hormone, and estrogen-dependent and estrogen-independent breast cancer cells (MCF-7 and MDA-MB-231) with an estrogen hormone to investigate cell proliferation, viability, adhesion and hormone expression. Moreover, the present study identifies the expression of *piRNA-651 (piR-651)* and *piRNA-823 (piR-823)*, which are among the piRNAs thought to be effective in gender-dependent cancers.

## Materials and Methods

### Cell Culture and Concentration of Androgen and Estrogen Hormones

Prostate cancer cell lines (LNCaP and PC-3) and breast cancer cell lines (MCF-7 and MDA-MB-231) were purchased from the American Type Culture Collection (ATCC, Washington D.C., USA), and the cells were incubated at 37°C with 5% CO_2_ in Dulbecco’s Modified Eagle’s Medium (DMEM; Gibco, United Kingdom) containing 1% penicillin/streptomycin (Gibco, UK) and 10% foetal bovine serum (FBS) (Gibco, UK). Before hormone treatment, the breast and prostate cancer cells were incubated in 6-well plates (Greiner, Germany) to make sure the cells grew to 80% confluence. Estrogen (E2257) and androgen (A0887) were obtained from Sigma (St. Louis, USA). To observe the appropriate concentration, hormones with certain concentration ranges were used to treat cells according to the instructions of the manufacturer. Using the XTT method (Biological Industries, Israel), the most proliferative concentration and hours were determined to be the 24^th^, 48^th^ and 72^nd^ hours. By deciding on the optimal concentration of hormones and optimal time, viability, proliferation, adhesion and piRNA expression, assays were applied to these specified conditions.

### Viability, Proliferation and Adhesion

The cell viability rate was determined using the trypan blue method. Hormone treated cells were exposed to trypan blue (Gibco, UK) after being trypsinized from a flask. The live and dead cells were counted using the Thoma Slide (Marienfeld, Germany).

The XTT Kit (Biological Industries, Israel) was used to determine the impacts of hormones on the proliferation of breast and prostate cancer cell lines. In brief, the cells were plated into 96-well plates (Greiner, Germany) and, after optimal hormone treatment, XTT was pipetted to all wells and cultured at 37°C with 5% CO_2_ for 2 hours. The microplate spectrophotometer (Lab Systems, Finland)) was used to determine the absorbance at 450nm.

The XTT Kit (Biological Industries, Israel) was again used, this time to determine the impacts of hormones on the adhesion of breast and prostate cancer cell lines, and, as before, the cells were plated into 96-well plates (Greiner, Germany). After optimal hormone treatment, the wells were flushed three times with phosphate-buffered saline (PBS; Sigma, Saint Louis, USA). XTT was then pipetted to all wells and cultured at 37°C with 5% CO_2_ for 2 hours, and the microplate spectrophotometer (Lab Systems, Finland)) was used to determine the absorbance at 450nm.

### Real Time Polymerase Chain Reaction (RT-PCR)

Total RNA was isolated from cells using the Paris Total RNA Isolation Kit (Ambion, Carlsbad, USA) in accordance with the instructions of the manufacturer. The RNAs isolated from the hormone treated cells were converted to cDNA through a reverse transcription (Bioneer, Alameda, USA). Primer sets for the amplification of *piR–651*, *piR–823* and *Glyseraldehide-3-phosphate dehydrogenase* (*GAPDH)* were designed and supplied by Alpha DNA, Montreal, Quebec. RT-PCR was carried out inside a Strategene MxPro3000 (Strategene, UK). *GAPDH* (Alpha DNA, Montreal, Quebec) was used as an internal control, and the expression of *piR–651* and *piR–823* was normalized in line with the expression of *GAPDH*. Gene expression changes were quantified using the delta-delta CT method. The forward and reverse primer sequences used in RT-PCR are shown in Table 1 [10,25,27].

**Table 1:** The forward and reverse primer sequences used in RT-PCR.

Primer	Forward Primer	Reverse Primer
**piR-651**	5′-AGAGAGGGGCCCGTGCCTTG-‘3	3’-CCAGTCTCAGGGTCCGAGGTATTC-‘5
**piR-823**	5′-AGCGTTGGTGGTATAGTGGT-‘3	3’-CCAGTCTCAGGGTCCGAGGTATTC-‘5
**GAPDH**	5’-CGAGGGGGGAGCCAAAAGGG-‘3	3’-GAAACTGCGACCCCGACCGT-‘5

### Statistical Analysis

A normal distribution of the continuous variables was enabled using the Kolmogorov-Smirnov suitability test. Comparisons between groups of normally distributed variables were evaluated using One-Way variance analysis (ANOVA). The Tukey HSD test was used for multiple comparisons. Comparisons between groups of variables which were not normally distributed were evaluated using the Kruskal-Wallis test. Multiple comparisons of these groups were evaluated using the Dunn test, while normally distributed piRNA values were compared using the Student t test. All analyses were carried out using the IBM SPSS Statistics 21.0 software package (S1–S10 Files). The obtained data were indicated as mean ± standard deviation (sd). In the figures, only mean values have been shown.

## Results

### Determining the Optimal Hormone Concentrations on Prostate and Breast Cancer Cell Lines

LNCaP androgen-dependent prostate cancer cells were treated with androgen at concentrations of 200nM, 100nM, 50nM, 25nM, 10nM, 1nM, and 0.1nM. Ethanol was used to dissolve the androgen and estrogen hormones in the medium, an additional ethanol group being formed for this reason. In the measurement carried out 24 hours after treatment with the hormone, statistically significant increases were observed in the 10nM androgen group (847229±19949) (P < 0.05; data not shown) and the 1nM androgen group (915657±25069) (P < 0.01; data not shown) when compared with the control group (156729±22278). The PC-3 cells were treated with 10nM, 1nM and 0.1nM androgen, and, when compared with the control group (41961±907), there was a statistically significant decrease in the 1nM androgen group (25990±5334) (P < 0.05; data not shown). The MCF–7 cells were treated with 10nM, 1nM and 0.1nM estrogen, but no statistically significant difference was found between the groups (P >0.05; data not shown). The MDA-MB–231 cells were treated with 10nM, 1nM and 0.1nM estrogen, but no statistically significant difference was found between the groups (P >0.05; data not shown).

### The Effects of Optimal Hormone Concentrations on Proliferation, Viability, and Adhesion of Prostate and Breast Cancer Cells

When compared with the control group (156728.57±58943.07), a significant increase in the number of cells in the 1nM androgen group (915657.14±66326.66) was detected in the LNCaP cells (P < 0.001). While cells were detected in the control group (41961.43±2398.41) and in the 10nM androgen group (40561.43±5547.29) of PC-3 cells, but no statistically significant difference was found between the groups (P >0.05). In the MCF-7 cells, an increase was detected in the proliferation of the 10nM estrogen group (97704.71±7623.37) when compared with the ethanol group (82252.29±14261.99) (P < 0.05). In the MDA-MB-231 cells, white cells were detected in the control group (20816.71±7496.41) and 1nM estrogen group (32489.29±10916.76) (Table 2) (P <0.05).

**Table 2:** The Proliferation Values of the LNCaP, PC-3, MCF-7 and MDA-MB-231 cells.

PROLIFERATION				
	Group	Mean ±sd	p value	Multiple comparisons and p value
**LNCaP**	CONTROL(1)	156728.57±58943.07	0.001†	1–3 p = 0.003††
	ETHANOL(2)	166157.14±68929.74		2–3 p = 0.008††
	1 nM ANDROGEN(3)	915657.14±66326.66		
**PC-3**	CONTROL(1)	41961.43±2398.41	0.066*	-
	ETHANOL(2)	34061.43±9023.99		
	10 nM ANDROGEN(3)	40561.43±5547.29		
**MCF-7**	CONTROL(1)	83514.29±9812.17	0.029*	2–3 p = 0.041**
	ETHANOL(2)	82252.29±14261.99		
	10 nM ESTROGEN(3)	97704.71±7623.37		
**MDA-MB-231**	CONTROL(1)	20816.71±7496.41	0.048†	1–3 p = 0.018††
	ETHANOL(2)	27525±7442.84		
	1 nM ESTROGEN(3)	32489.29±10916.76		

*: One-Way ANOVA,

**: Tukey HSD Test,

^†^:Kruskal-Wallis Test,

^††^:Dunn Test.

All obtained data were compared with the control group (n = 7 for each cell line)

In the LNCaP cells, a statistically significant increase was detected in the viable cell count percentage (86.75±6.84) of the 1nM androgen group when compared with the control (80.88±9.92) and ethanol groups (68±9.21) (P < 0.001). In the PC-3 cells, while the viable cell count percentage was detected to have increased in the 10nM androgen group (78.75±20.83) when compared to the ethanol group (73.88±19.48), no statistically significant difference was found between the groups (P >0.05). It was observed that the treatment of the MCF-7 cells with estrogen increased the viable cell count percentage of the 10nM estrogen group (86.75±9.54) when compared with the control (82.13±12.41) and ethanol (71.88±8.27) groups (P < 0.05). In the MDA-MB-231 cells, while the viable cell count % was detected to have increased in the 1nM estrogen group (86.13±15.41) when compared with the control (72.88±29.53) and ethanol (77±21.58) groups, no statistically significant difference was found between the groups (Table 3) (P >0.05).

**Table 3:** The Cell Viability Rate of LNCaP, PC-3, MCF-7 and MDA-MB-231.

VIABILITY				
	Group	Mean±sd	p value	Multiple comparisons and p value
**LNCaP**	CONTROL(1)	80.88±9.92	0.001*	1–2 p = 0.020**
	ETHANOL(2)	68±9.21		1–3 p = 0.001**
	1 nM ANDROGEN(3)	86.75±6.84		
**PC-3**	CONTROL(1)	86.13±15.41	0.406†	-
	ETHANOL(2)	73.88±19.48		
	10 nM ANDROGEN(3)	78,75±20.83		
**MCF-7**	CONTROL(1)	82.13±12.41	0.025*	2–3 p = 0.022**
	ETHANOL(2)	71.88±8.27		
	10 nM ESTROGEN(3)	86.75±9.54		
**MDA-MB-231**	CONTROL(1)	72.88±29.53	0.588†	-
	ETHANOL(2)	77±21.58		
	1 nM ESTROGEN(3)	86.13±15.41		

*: One-way ANOVA,

**: Tukey HSD test,

^†^: Kruskal-Wallis test.

All obtained data were compared with the control group (n = 8 for each cell line)

In the LNCaP cells, it was observed that adhesion decreased in the 1nM androgen group (0.79±0.12) when compared with the control group (0.93±0.09) (P < 0.05), while in the PC-3 cells, a statistically significant increase was detected in the adhesion in ethanol (0.86±0.06) and the 10nM androgen group (0.84±0.06) when compared with the control group (0.74±0.05) (P < 0.01). In the MCF-7 cells, while it was observed that adhesion increased in the 10nM estrogen group (1.18±0.58) when compared to the control (0.69±0.17) and ethanol (0.66±0.12) groups, no statistically significant difference was found between the groups (P >0.05). In the MDA-MB–231 cells, it was observed that adhesion decreased in the 1nM estrogen group (0.69±0.05) when compared to the control (0.74±0.06) and ethanol (0.70±0.03) groups, but no statistically significant difference was found between the groups (Table 4) (P >0.05). Based on these results, it was determined that adhesion decreased in the LNCaP cells treated with androgen while it increased in PC-3 cells.

**Table 4:** The Adhesion Values of LNCaP, PC-3, MCF-7 and MDA-MB-231 cells.

ADHESION				
	Group	Mean±sd	p value	Multiple comparisons and p value
**LNCaP**	CONTROL(1)	0.93±0.09	0.018†	1–3 p = 0.048††
	ETHANOL(2)	0.90±0.07		2–3 p = 0.037††
	1 nM ANDROGEN(3)	0.79±0.12		
**PC-3**	CONTROL(1)	0.74±0.05	0.001*	1–2 p = 0.002**
	ETHANOL(2)	0.86±0.06		1–3 p = 0.008**
	10 nM ANDROGEN(3)	0.84±0.06		
**MCF-7**	CONTROL(1)	0.69±0.17	0.077†	-
	ETHANOL(2)	0.66±0.12		
	10 nM ESTROGEN(3)	1.18±0.58		
**MDA-MB-231**	CONTROL(1)	0.74±0.06	0.2*	-
	ETHANOL(2)	0.70±0.03		
	1 nM ESTROGEN(3)	0.69±0.05		

*: One-way ANOVA,

**: Tukey HSD test,

^†^: Kruskal-Wallis test,

^††^: Dunn test.

All obtained data were compared with the control group (n = 7 for each cell line)

### Determining Expressions of piR-651 and piR-823 in Optimal Hormone Concentrations for Prostate and Breast Cancer Cells

**Fig 1:**
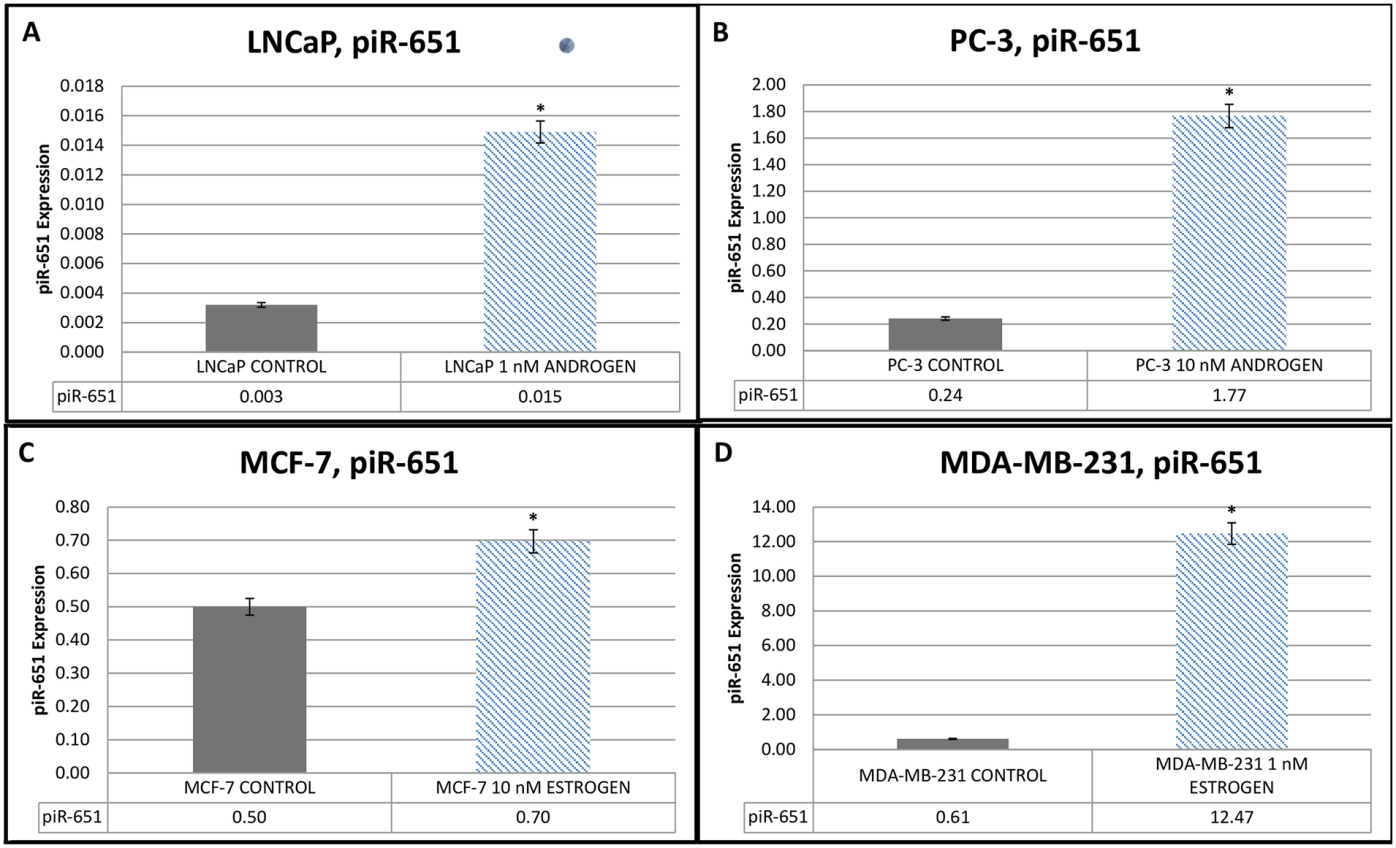
piR-651 Expressions of androgen dependent and independent prostate cancer cell lines and estrogen-dependent and estrogen-independent breast cancer cell lines. (A) piR-651 Expression of androgen-dependent LNCaP cells before and after 1nM androgen hormone treatment. (B) piR-651 Expression of androgen-independent PC-3 cells before and after 10nM androgen hormone treatment. (C) piR-651 Expression of estrogen-dependent MCF-7 cells before and after 10nM estrogen hormone treatment. (D) piR-651 Expression of estrogen-independent MDA-MB-231 cells before and after 1nM estrogen hormone treatment. All obtained data were compared with the control group *P < 0.001. (n = 7 for each cell line).

Statistically significant increases in the expression levels of *piR-651* were observed in the 1nM androgen group (0.015±0.0002) when compared with the control group (0.003±0.0002) for the LNCaP cells (Fig 1A), in the 10nM androgen group (1.77±0.0002) when compared with the control group (0.24±0.0002) for the PC-3 cells (Fig 1B), in the 10nM estrogen group (0.7±0.0002) when compared with the control group (0.5±0.0002) for the MCF–7 cells (Fig 1C), and in the 1nM estrogen group when compared with the control group (0.61±0.0002) for the MDA-MB–231 cells (Fig 1D) (P < 0.001).

**Fig 2:**
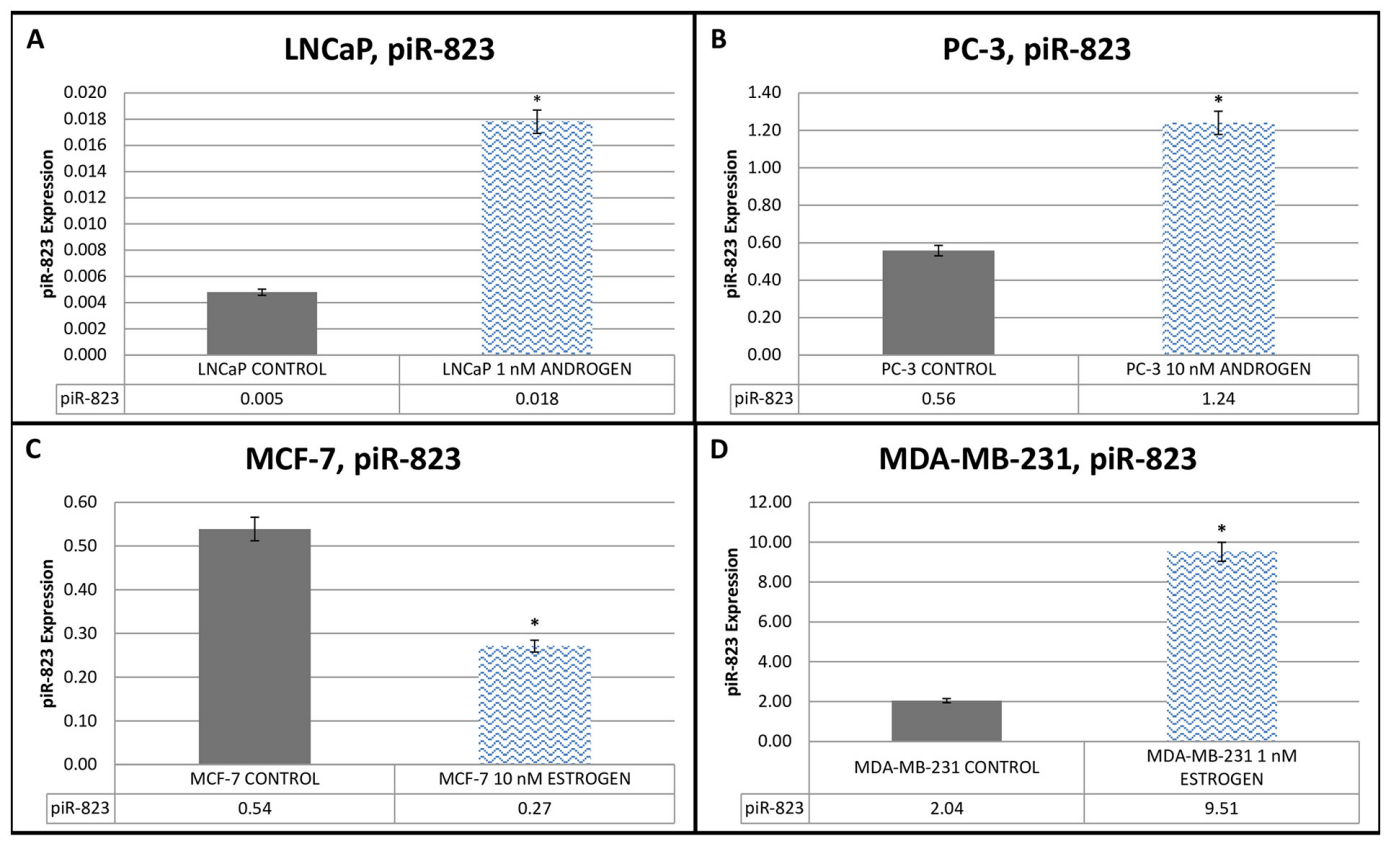
piR-823 Expression of androgen-dependent and androgen-independent prostate cancer cell lines and estrogen-dependent and estrogen-independent breast cancer cell lines. (A) piR-823 Expression of androgen-dependent LNCaP cells before and after 1nM androgen hormone treatment. (B) piR-823 Expression of androgen-independent PC-3 cells before and after 10nM androgen hormone treatment. (C) piR-823 Expression of estrogen-dependent MCF-7 cells before and after 10nM estrogen hormone treatment. (D) piR-823 Expression of estrogen-independent MDA-MB-231 cells before and after 1nM estrogen hormone treatment. All obtained data were compared with the control group *P < 0.001. (n = 7 for each cell line).

Statistically significant increases in the expression levels of *piR-823* were observed in the 1nM androgen group (0.018±0.0002) when compared with the control group (0.005±0.0002) for the LNCaP cells (Fig 2A), in the 10nM androgen group (1.24±0.0002) when compared with the control group (0.56±0.0002) for the PC-3 cells (Fig 2B), and in the 1nM estrogen group (9.51±0.0002) when compared with the control group (2.04±0.0002) for the MDA-MB–231 cells (Fig 2D) (P < 0.001). Contrary to the other cell lines, a statistically significant decrease was observed in the level of *piR-823* expression in the 10nM estrogen group (0.27±0.0002) when compared with the control group (0.54±0.0002) for the MCF–7 cells (Fig 2C) (P < 0.001).

## Discussion

In recent times, studies investigating the relationship between piRNAs and cancer have been increasing rapidly. In our study, the effects of hormones in a medium containing cancer cells on piRNAs were determined. The literature review which was carried out to determine the hormone concentrations with which the cancer cells would be treated yielded varied information. In previous studies on an androgen-dependent prostate cancer cell line LNCaP, the cells were treated with androgen at concentrations ranging from 1nM to 10nM. [7,28–31]. In our study, we detected that the concentration at which androgen increased cell proliferation the greatest amount was 1nM at the 24^th^ hour for the androgen-dependent prostate cancer cell line LNCaP. Considering the possibility of interaction with hormones, we did not use serum in our study. The absence of serum causes a shortage in the cells and induces other mechanisms [32–38]. Moreover, several previous studies reported that ethanol in cancer cells induced proliferation by increasing the cyclic AMP (cAMP) level after a certain period of time [39–43]. For this reason, the duration of a serum-free trial was discovered to be 24 hours. While we were planning the study, we wanted to compare the piRNAs which have known expression profiles in other cancers. Therefore, we gave priority to the comparison of piR-651 and piR-823 expressions in breast and prostate cancer when androgen and estrogen hormones were both present and absent. On the other hand, we also wanted to observe the effect of androgen and estrogen on the viability, proliferation and adhesion of breast and prostate cancer cell lines. These assays concerning cell behaviour can be carried out in further studies.

Androgen-independent cancer cells were treated with an androgen hormone at concentrations ranging from 10^−13^ to 10^-7^nM [44–46]. However, since PC-3 prostate cancer cells are androgen-independent cells, they did not present significant responses to the androgen used in the studies. Based on the data we obtained, we determined that the concentration at which the highest proliferation for PC-3 cells treated with androgen was observed to be 10nM at the 24^th^ hour.

In the studies investigating the effects of estrogen on MCF–7 and MDA-MB–231 breast cancer cells, the cells were treated with androgen hormones at various concentrations and the effects of this treatment on cell proliferation was investigated. The studies showed that the optimum concentration of estrogen was between 0.01 and 10nM for MCF-7 cells [38,47–51], and the conclusion was that cell proliferation was equal at each one of a variety of concentrations for MDA-MB–231 cells and that estrogen did not have any effects on these cells [35,49,50]. Based on the data we obtained, we determined that the concentration at which the highest proliferation for MCF-7 cells treated with estrogen was observed to be 10nM at the 24^th^ hour. For the estrogen-independent cancer cell line MDA-MB–231, 1nM at the 24^th^ hour was determined to be the concentration of the highest proliferation.

In order to determine the effects of the substances and/or chemicals with which the cells are treated on the cell count, cell proliferation assays are carried out in the studies on the diagnosis or treatment of most diseases. According to previous studies, the androgen hormone increases the survival and proliferation of androgen-dependent prostate cancer cells [28,46,52,53]. The studies investigating the prostate cancer cell line LNCaP argue that tumor development is correlated with androgen levels, and report that cell proliferation depends on the androgen level [7,28,46]. In our study, increases were observed in the proliferation and viable cell count of LNCaP cells treated with 1nM androgen. The studies determined that androgen is not effective in the proliferation and viability of PC-3 cells [44,46,54]. As with these studies, we also determined that the androgen with which the cells were treated was not effective for PC-3 cells in terms of proliferation and viability.

The proliferation, development, transcriptional and translational mechanisms of breast cancer cells are regulated by many signal molecules and cell cycles, among which hormones also play important roles. Various studies have reported that estrogen at various concentrations caused an increase in the proliferation and viability of the estrogen-dependent breast cancer cell line MCF-7 [33,34,37,38,48,51,55]. Our study shows that 10nM estrogen may be effective in the proliferation and viability of MCF–7 cells. MDA-MB–231 cells are estrogen-independent cells and studies have revealed that estrogen at various concentration had effects on the proliferation and viability of the cells [36,56,57]. The data obtained in our study shows that estrogen does not affect the proliferation and viability of MDA-MB–231 cells.

Adhesion is important both pathologically and physiologically in cancer and inflammatory diseases. The effectiveness of anti-cancer drugs administered to tumor tissue is affected by cell adhesion and the density of the tumor cells [3]. The application of androgen into the medium increased metastatic characteristics and reduced adhesion (LNCaP, LNCaP TβRII and PC-3) [52]. Contrary to these studies, it was determined that 10nM androgen increased the adhesion of PC-3 cells. Sapino A. et al. found increases in the adhesion plaques of MCF-7 cells treated with estrogen, as well as increases in the number of adhesion-specific structures [58]. Based on the data we obtained, the estrogen with which both breast cancer cell lines were treated had no statistically significant effect on the adhesion of cells.

The level of piRNAs is high in both the normal gamete stem cells and cancer cells. A study by Yang Q. et al. identified 770 known miRNA and 5 new miRNA lines, as well as 20121 piRNA lines in healthy human testes. In this study, common line matches were found between piRNAs and miRNAs by using the advanced sequencing (Next Generation Sequencing—NGS) method. The study by Yang et al. showed that miRNAs and piRNAs act as regulators in spermatogenesis, and it is a useful source for studies in this field [59]. In their study, Kang et al. administered testosterone hormone to the testes of healthy male rats and measured piRNA expressions at certain time intervals. They eventually determined that testosterone increased the expression of the PIWI protein and piRNA in the testis [60].

In their study, Chu H. et al. investigated the single nucleotide polymorphism (SNP) of the sites containing piRNA clusters in the development of colorectal cancer. They determined that individuals that have the polymorphism of *piR-01551* and the long non-coding RNA site *lnc00964-3* had a high risk of developing colorectal cancer [61]. The results of microarray and other functionality assays of hepatocellular carcinoma revealed that the expression of the gene site *piR-Hep1* increased by 46.6% in hepatocellular carcinoma tissue when compared with healthy tissue. In the same study, it was argued that silencing of the gene site piR-Hep1 prevented the cancer cells from proliferating, migrating, and attacking the healthy tissue [62]. Another study on bladder cancer, however, showed that piRNAs play a role in tumor development [63]. Hashim et al suggested that expression of at least three piRNAs (DQ597945, DQ570994 and DQ598651) was found to be significantly different in estrogen positive and negative breast cancer cell lines. In their study, it was also found that eight piRNAs deregulated in the breast cancer tumors’ encode proteins involved in key cancer cell functions. All of their data showed that piRNAs represent a new perspective in the identification of estrogen levels in hormone responsive breast cancer types [64]. In another study, it was suggested that piRNA expression profiles might be evidence supporting a potentially tissue-specific role for piRNAs in both tumor and non-malignant somatic tissues that may be clinically relevant. Furthermore, in this study, it was also shown that piRNA expression can delineate clinical features, such as histological subgroups, stages, and survival. It was indicated that 6260 piRNA transcriptome profiles were summarized according to tumor and non-malignant tissue types from 11 organs (bladder, breast, colon, head and neck, kidney, lung, ovaries, prostate, stomach, thyroid, and uterine corpus) [65].

It is postulated that the *piR-651* and *piR-823* that we studied take part in the development of various cancers, especially gastric cancer [10,25,27,66]. In their study, Yan H. et al. detected an increase in the expression of *piR-823* in the tissue samples and cell lines of patients with multiple myeloma, and it was also observed that silencing of *piR-823* in multiple myeloma cell lines resulted in dysfunction of the elements playing roles in the expression of protein associated with apoptosis and in the regulation of the cell cycle. Additionally, it was determined that *piR–823* was directly associated with *DNMT3A and 3B* de novo DNA methyltransferases [67]. As with the data obtained by Yan H. et al. for multiple myeloma, it was found that silencing of *piR-823* in the therapeutic xenograft models effectively inhibited tumor growth [67]. Another study, on the other hand, reported that *piR-823* expression decreased in gastric cancer and displayed tumor suppressing properties both *in vivo* and *in vitro* [27]. The *piR-823* expression that displays different properties (heterogenic, tumor suppressing or oncogenic) in different cancer types is thought to play a dual role in tumor formation. The data we obtained shows that androgen increases the *piR-823* expression in LNCaP and PC-3 prostate cancer cells. The treatment of MDA-MB–231 cells with estrogen increases the *piR–823* expression. Increasing the hormone levels in cancer cells by external administration of the hormones into these cells revealed that the *piR-823* expression displayed oncogenic properties in prostate cancer cell lines and malignant estrogen-independent breast cancer lines, and that this property increased with the increase in hormone levels. The treatment of MCF–7 breast cancer cells with estrogen reduces the *piR–823* expression. This result shows that *piR-823* may have a different kind of expression in benign estrogen-dependent breast cancer cells treated with hormones. It was observed that therapy with the external administration of estrogen hormone had a positive effect in benign estrogen-dependent MCF-7 cancer cells and caused a decrease in the normally high *piR-823* expression.

In our study, the piRNA site is thought to be the one that plays a role in *piR-651* cancer development. Previous studies showed that the *piR-651* expression increased in various tumors, including gastric, colon, lung and breast tumors, when compared to healthy tissues [10]. Additionally, there is clinical data supporting the notion that increased expression of the *piR-651* and PIWI protein during cancer development may play a role in a variety of cancer types, including gastric, pancreas and oesophageal cancer [68–70]. A recent study argued that *piR–651* may play a part in the metastasis of gastric tumor cells to the lymph nodes [10]. It was observed that cancer cell growth decreased when *piR-651* was silenced in gastric cancer [25]. These studies are among the studies carried out to determine piRNA expression levels in cancer [71]. Moreover, it was also investigated whether the *piR-651* expression increased in mesothelioma, hepatocellular, and cervical cancer [10].

Upon the determination of the concentrations at which the externally administered hormones increased cell proliferation the most, piRNA expressions were evaluated in these cells. It was observed that the viability was also at its highest level at the highest proliferation concentrations of the externally administered hormones while adhesion was reduced. On the contrary, a decrease in adhesion was detected in MDA-MB-231 cells. While it was observed that the expression of *piR-651* and *piR-823* increased upon administration, only the *piR-823* expression was detected to have decreased in the estrogen-dependent breast cancer cell line MCF-7.

This study showed that piRNAs, which are becoming increasingly important today, may be affected by hormones. In general, it was observed that cell proliferation and viability increased with androgen or estrogen treatment and that cell adhesion decreased in cells other than PC-3 cells. It was detected that the *piR-651* expression increased in LNCaP and PC-3 prostate cancer cells treated with androgen and MCF-7 and MDA-MB-231 breast cancer cells treated with estrogen. The results of this study show that the increase in the expression of *piR-651* and *piR-823* during gonad development and in cancer may be associated with hormone levels in some cancer cells, and that these piRNA expressions vary depending on the hormone level and cancer cell type in the microenvironment of the cancer. We think that our study will contribute greatly to future studies on piRNAs and may be a source for the consideration of these properties in hormone therapies for some cancer types. More molecular studies are needed in order to obtain detailed information about certain results for the mechanisms of piRNAs in cancer.

## Supporting Information

S1 File Statistically determined proliferation values of LNCAP cells. (PDF)

S2 File Statistically determined proliferation, adhesion and viability values of PC3 cells. (PDF)

S3 File Statistically determined adhesion and proliferation values of MCF-7 cells. (PDF)

S4 File Statistically determined adhesion and proliferation values of MDA-MB-231 cells. (PDF)

S5 File Statistically determined viability values of LNCAP cells. (PDF)

S6 File Statistically determined viability values of MCF-7 cells. (PDF)

S7 File Statistically determined viability values of MDA-MB-231 cells. (PDF)

S8 File Statistically determined adhesion values of LNCAP cells. (PDF)

S9 File Statistically expressions of piR651 and piR823 in MCF-7, MDA-MB-231 and LNCAp cells. (PDF)

S10 File Statistically determined expressions of piR651 and piR823 in PC-3 cells. (PDF)
