# Supplementary material for: Estrogen and Androgen Hormone Levels Modulate the Expression of PIWI Interacting RNA in Prostate and Breast Cancer
Source: PLoS One. 2016 Jul 14;11(7):e0159044. doi: 10.1371/journal.pone.0159044 (PMC4944994; doi:10.1371/journal.pone.0159044)
Supplement: S1 File — (PDF) [file pone.0159044.s001.pdf]

# Explore

## grup

**Tests of Normality**

| | grup | Kolmogorov-Smirnov[a] | | | Shapiro-Wilk | | |
|---|---|---|---|---|---|---|---|
| | | Statistic | df | Sig. | Statistic | df | Sig. |
| LNCAP_Pro | Normal | ,372 | 7 | ,004 | ,759 | 7 | ,016 |
| | Etanol | ,377 | 7 | ,003 | ,716 | 7 | ,006 |
| | Androjen1 nM | ,162 | 7 | ,200* | ,980 | 7 | ,959 |

*. This is a lower bound of the true significance.

a. Lilliefors Significance Correction

# Nonparametric Tests

**Descriptives**

LNCAP_Pro

| | N | Mean | Std. Deviation | Std. Error | Minimum | Maximum |
|---|---|---|---|---|---|---|
| Normal | 7 | 156728,5714 | 58943,07181 | 22278,38707 | 111800,00 | 269800,00 |
| Etanol | 7 | 166157,1429 | 68929,74339 | 26052,99414 | 119800,00 | 302800,00 |
| Androjen1 nM | 7 | 915657,1429 | 66326,66274 | 25069,12213 | 823800,00 | 1,02E+006 |
| Total | 21 | 412847,6190 | 369502,5678 | 80632,07078 | 111800,00 | 1,02E+006 |

**Notes**

| | | |
|---|---|---|
| Output Created | | 26-MAY-2015 11:05:35 |
| Comments | | |
| Input | Data | C:\Users\pc\Desktop\LNCAP_Pro.sav |
| | Active Dataset | DataSet0 |
| | Filter | <none> |
| | Weight | <none> |
| | Split File | <none> |
| | N of Rows in Working Data File | 21 |
| Syntax | | NPTESTS /INDEPENDENT TEST (LNCAP_Pro) GROUP (grup) KRUSKAL_WALLIS (COMPARE=PAIRWISE) /MISSING SCOPE=ANALYSIS USERMISSING=EXCLUDE /CRITERIA ALPHA=0.05 CILEVEL=95. |
| Resources | Processor Time | 00:00:00,17 |
| | Elapsed Time | 00:00:00,28 |

## Hypothesis Test Summary

| | Null Hypothesis | Test | Sig. | Decision |
|---|---|---|---|---|
| 1 | The distribution of LNCAP_Pro is the same across categories of grup. | Independent-Samples Kruskal-Wallis Test | ,001 | Reject the null hypothesis. |

Asymptotic significances are displayed. The significance level is ,05.

## Pairwise Comparisons of grup

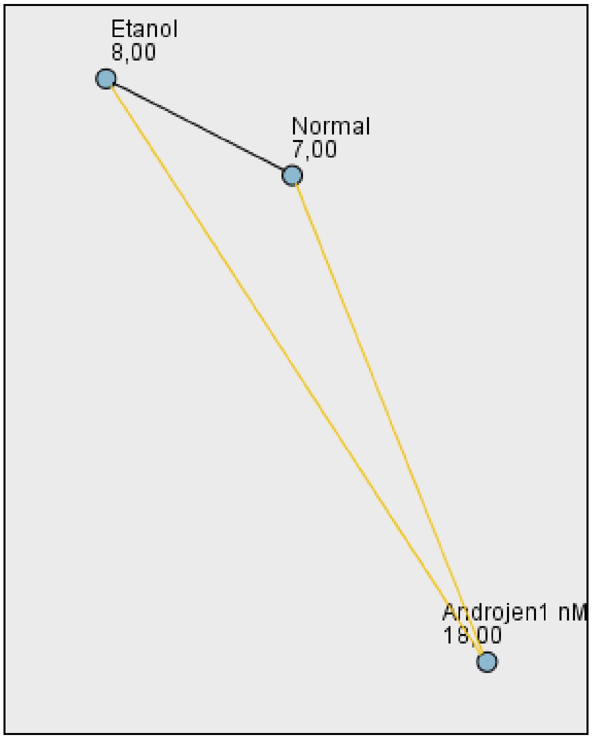


Each node shows the sample average rank of grup.

| Sample1-Sample2 | Test Statistic | Std. Error | Std. Test Statistic | Sig. | Adj.Sig. |
|---|---|---|---|---|---|
| **Normal-Etanol** | -1,000 | 3,317 | -,302 | ,763 | 1,000 |
| **Normal-Androjen1 nM** | -11,000 | 3,317 | -3,317 | ,001 | ,003 |
| **Etanol-Androjen1 nM** | -10,000 | 3,317 | -3,015 | ,003 | ,008 |

Each row tests the null hypothesis that the Sample 1 and Sample 2 distributions are the same.
Asymptotic significances (2-sided tests) are displayed. The significance level is ,
[illegible]

**Notes**

| | | |
|---|---|---|
| Output Created | | 26-MAY-2015 11:06:09 |
| Comments | | |
| Input | Data | C:\Users\pc\Desktop\LNCAP_Pro.sav |
| | Active Dataset | DataSet0 |
| | Filter | <none> |
| | Weight | <none> |
| | Split File | <none> |
| | N of Rows in Working Data File | 21 |
| Missing Value Handling | Definition of Missing | User-defined missing values are treated as missing. |
| | Cases Used | Statistics for each analysis are based on cases with no missing data for any variable in the analysis. |
| Syntax | | ONEWAY LNCAP_Pro BY grup /STATISTICS DESCRIPTIVES /MISSING ANALYSIS /POSTHOC=TUKEY ALPHA(0.05). |
| Resources | Processor Time | 00:00:00,03 |
| | Elapsed Time | 00:00:00,03 |
