# Supplementary material for: Estrogen and Androgen Hormone Levels Modulate the Expression of PIWI Interacting RNA in Prostate and Breast Cancer
Source: PLoS One. 2016 Jul 14;11(7):e0159044. doi: 10.1371/journal.pone.0159044 (PMC4944994; doi:10.1371/journal.pone.0159044)
Supplement: S2 File — (PDF) [file pone.0159044.s002.pdf]

Explore

Grup

Tests of Normality

	Grup	Kolmogorov-Smirnov[a]			Shapiro-Wilk	
		Statistic	df	Sig.	Statistic	df
PC3_Adezyon	Normal	,198	7	,200*	,912	7
	Etanol	,162	7	,200*	,958	7
	Androjen 10 nM	,202	7	,200*	,923	7
PC3_Prolirefasyon	Normal	,210	7	,200*	,954	7
	Etanol	,216	7	,200*	,944	7
	Androjen 10 nM	,263	7	,156	,900	7

Tests of Normality

	Grup	Shapiro-...
		Sig.
PC3_Adezyon	Normal	,411
	Etanol	,797
	Androjen 10 nM	,489
PC3_Prolirefasyon	Normal	,762
	Etanol	,676
	Androjen 10 nM	,333

*. This is a lower bound of the true significance.

a. Lilliefors Significance Correction

Oneway

Notes

Output Created		29-MAY-2015 14:28:08
Comments		
Input	Data	C:\Users\pc\Desktop\PC3 pro ad canlılık.sav
	Active Dataset	DataSet0
	Filter	<none>
	Weight	<none>
	Split File	<none>
	N of Rows in Working Data File	24
Missing Value Handling	Definition of Missing	User-defined missing values are treated as missing.
	Cases Used	Statistics for each analysis are based on cases with no missing data for any variable in the analysis.
Syntax		ONEWAY PC3_Adezyon PC3_Prolirefasyon BY Grup /STATISTICS DESCRIPTIVES /MISSING ANALYSIS /POSTHOC=TUKEY ALPHA(0.05).
Resources	Processor Time	00:00:00,02
	Elapsed Time	00:00:00,01

Descriptives

		N	Mean	Std. Deviation	Std. Error
PC3_Adezyon	Normal	7	,738286	,0519927	,0196514
	Etanol	7	,862857	,0569925	,0215411
	Androjen 10 nM	7	,840714	,0591403	,0223529
	Total	21	,813952	,0769828	,0167990
PC3_Prolirefasyon	Normal	7	41961,4286	2398,41217	906,51459
	Etanol	7	34061,4286	9023,98919	3410,74732
	Androjen 10 nM	7	40561,4286	5547,28591	2096,67700
	Total	21	38861,4286	6915,64479	1509,11742

Descriptives

		Minimum	Maximum
PC3_Adezyon	Normal	,6390	,7960
	Etanol	,7940	,9620
	Androjen 10 nM	,7670	,9170
	Total	,6390	,9620
PC3_Prolirefasyon	Normal	37990,00	45790,00
	Etanol	20990,00	48490,00
	Androjen 10 nM	33090,00	50990,00
	Total	20990,00	50990,00

ANOVA

		Sum of Squares	df	Mean Square	F
PC3_Adezyon	Between Groups	,062	2	,031	9,816
	Within Groups	,057	18	,003	
	Total	,119	20		
PC3_Prolirefasyon	Between Groups	248780000,0	2	124390000,0	3,164
	Within Groups	707742857,1	18	39319047,62	
	Total	956522857,1	20		

ANOVA

		Sig.
PC3_Adezyon	Between Groups	,001
	Within Groups	
	Total	
PC3_Prolirefasyon	Between Groups	,066
	Within Groups	
	Total	

Multiple Comparisons

Tukey HSD

Dependent Variable	(I) Grup	(J) Grup	Mean Difference (I-J)	Std. Error	Sig.
PC3_Adezyon	Normal	Etanol	-,1245714*	,0299983	,002
		Androjen 10 nM	-,1024286*	,0299983	,008
	Etanol	Normal	,1245714*	,0299983	,002
		Androjen 10 nM	,0221429	,0299983	,744
	Androjen 10 nM	Normal	,1024286*	,0299983	,008
		Etanol	-,0221429	,0299983	,744
PC3_Prolirefasyon	Normal	Etanol	7900,00000	3351,71801	,073
		Androjen 10 nM	1400,00000	3351,71801	,909
	Etanol	Normal	-7900,00000	3351,71801	,073
		Androjen 10 nM	-6500,00000	3351,71801	,157
	Androjen 10 nM	Normal	-1400,00000	3351,71801	,909
		Etanol	6500,00000	3351,71801	,157

Multiple Comparisons

Tukey HSD

Dependent Variable	(I) Grup	(J) Grup	95% Confidence Interval	
			Lower Bound	Upper Bound
PC3_Adezyon	Normal	Etanol	-,201132	-,048011
		Androjen 10 nM	-,178989	-,025868
	Etanol	Normal	,048011	,201132
		Androjen 10 nM	-,054418	,098704
	Androjen 10 nM	Normal	,025868	,178989
		Etanol	-,098704	,054418
PC3_Prolirefasyon	Normal	Etanol	-654,1314	16454,1314
		Androjen 10 nM	-7154,1314	9954,1314
	Etanol	Normal	-16454,1314	654,1314
		Androjen 10 nM	-15054,1314	2054,1314
	Androjen 10 nM	Normal	-9954,1314	7154,1314
		Etanol	-2054,1314	15054,1314

*. The mean difference is significant at the 0.05 level.

Explore

Notes

Output Created		29-MAY-2015 14:29:28
Comments		
Input	Data	C:\Users\pc\Desktop\PC3 pro ad canlılık.sav
	Active Dataset	DataSet0
	Filter	<none>
	Weight	<none>
	Split File	<none>
	N of Rows in Working Data File	24
Missing Value Handling	Definition of Missing	User-defined missing values for dependent variables are treated as missing.
	Cases Used	Statistics are based on cases with no missing values for any dependent variable or factor used.
Syntax		EXAMINE VARIABLES=PC3_Canlılık BY Grup2<br>/PLOT NPPLOT<br>/PERCENTILES (5,10,25,50,75,90,95) HAVERAGE<br>/STATISTICS NONE<br>/MISSING LISTWISE...
Resources	Processor Time	00:00:00,64
	Elapsed Time	00:00:00,61

Grup2

Tests of Normality

	Grup2	Kolmogorov-Smirnov[a]			Shapiro-Wilk		
		Statistic	df	Sig.	Statistic	df	Sig.
PC3_Canlılık	Normal	,316	8	,018	,776	8	,016
	Etanol	,237	8	,200*	,889	8	,228
	Androjen 10 nM	,221	8	,200*	,847	8	,089

*. This is a lower bound of the true significance.

a. Lilliefors Significance Correction

Notes

Output Created		29-MAY-2015 14:30:14
Comments		
Input	Data	C:\Users\pc\Desktop\PC3 pro ad canlılık.sav
	Active Dataset	DataSet0
	Filter	<none>
	Weight	<none>
	Split File	<none>
	N of Rows in Working Data File	24
Missing Value Handling	Definition of Missing	User-defined missing values are treated as missing.
	Cases Used	Statistics for each analysis are based on cases with no missing data for any variable in the analysis.
Syntax		ONEWAY PC3_Canlılık BY Grup2<br>/STATISTICS DESCRIPTIVES<br>/MISSING ANALYSIS<br>/POSTHOC=TUKEY ALPHA(0.05).
Resources	Processor Time	00:00:00,00
	Elapsed Time	00:00:00,00

Descriptives

PC3_Canlılık

	N	Mean	Std. Deviation	Std. Error	95% Confidence Interval for Mean	
					Lower Bound	Upper Bound
Normal	8	86,1250	15,41277	5,44924	73,2396	99,0104
Etanol	8	73,8750	19,48213	6,88797	57,5875	90,1625
Androjen 10 nM	8	78,7500	20,83095	7,36485	61,3349	96,1651
Total	24	79,5833	18,61023	3,79880	71,7249	87,4417

Descriptives

PC3_Canlılık

	Minimum	Maximum
Normal	67,00	100,00
Etanol	40,00	100,00
Androjen 10 nM	50,00	100,00
Total	40,00	100,00

Nonparametric Tests

Notes

Output Created		29-MAY-2015 14:30:44
Comments		
Input	Data	C:\Users\pc\Desktop\PC3 pro ad canlılık.sav
	Active Dataset	DataSet0
	Filter	<none>
	Weight	<none>
	Split File	<none>
	N of Rows in Working Data File	24
Syntax		NPTESTS<br>/INDEPENDENT TEST (PC3_Canlılık) GROUP (Grup2) KRUSKAL_WALLIS (COMPARE=PAIRWISE)<br>/MISSING SCOPE=ANALYSIS USERMISSING=EXCLUDE<br>/CRITERIA ALPHA=0.05 CILEVEL=95.
Resources	Processor Time	00:00:00,17
	Elapsed Time	00:00:00,19

Hypothesis Test Summary

	Null Hypothesis	Test	Sig.	Decision
1	The distribution of PC3_Canlılık is the same across categories of Grup2.	Independent-Samples Kruskal-Wallis Test	,406	Retain the null hypothesis.

Asymptotic significances are displayed. The significance level is ,05.
