# Supplementary material for: Estrogen and Androgen Hormone Levels Modulate the Expression of PIWI Interacting RNA in Prostate and Breast Cancer
Source: PLoS One. 2016 Jul 14;11(7):e0159044. doi: 10.1371/journal.pone.0159044 (PMC4944994; doi:10.1371/journal.pone.0159044)
Supplement: S3 File — (PDF) [file pone.0159044.s003.pdf]

Explore

GRUP

Percentiles

		GRUP	Percentiles 25	50	75
Weighted Average (Definition 1)	MCF7ADEZYON	NORMAL	,5450	,6770	,6810
		ETANOL	,5780	,6330	,7010
		10 nM E	,6540	1,0810	1,4850
	MCF7PROL	NORMAL	76014,0000	83514,0000	89300,0000
		ETANOL	73871,0000	80300,0000	93157,0000
		10 nM E	88586,0000	97705,0000	104871,0000

Tests of Normality

		Kolmogorov-Smirnov[a]			Shapiro-Wilk		
	GRUP	Statistic	df	Sig.	Statistic	df	Sig.
MCF7ADEZYON	NORMAL	,378	7	,003	,756	7	,015
	ETANOL	,243	7	,200*	,814	7	,057
	10 nM E	,198	7	,200*	,889	7	,269
MCF7PROL	NORMAL	,135	7	,200*	,990	7	,994
	ETANOL	,214	7	,200*	,965	7	,860
	10 nM E	,170	7	,200*	,938	7	,621

*. This is a lower bound of the true significance.

a. Lilliefors Significance Correction

Descriptives

MCF7ADEZYON

	N	Mean	Std. Deviation	Std. Error	95% Confidence Interval for Mean Lower Bound	Upper Bound
NORMAL	7	,6899	,16632	,06286	,5360	,8437
ETANOL	7	,6646	,12072	,04563	,5529	,7762
10 nM E	7	1,1750	,57733	,21821	,6411	1,7089
Total	21	,8431	,41303	,09013	,6551	1,0312

Descriptives

MCF7ADEZYON

	Minimum	Maximum
NORMAL	,54	1,04
ETANOL	,56	,92
10 nM E	,62	2,29
Total	,54	2,29

Nonparametric Tests

Hypothesis Test Summary

	Null Hypothesis	Test	Sig.	Decision
1	The distribution of MCF7ADEZYON is the same across categories of GRUP.	Independent-Samples Kruskal-Wallis Test	,077	Retain the null hypothesis.

Asymptotic significances are displayed. The significance level is ,05.

Oneway

Descriptives

MCF7PROL

	N	Mean	Std. Deviation	Std. Error	95% Confidence Interval for Mean	
					Lower Bound	Upper Bound
NORMAL	7	83514,2857	9812,17313	3708,65285	74439,5391	92589,0323
ETANOL	7	82252,2857	14261,98530	5390,52376	69062,1493	95442,4222
10 nM E	7	97704,7143	7623,36813	2881,36232	90654,2747	104755,1539
Total	21	87823,7619	12604,59303	2750,54770	82086,2199	93561,3039

Descriptives

MCF7PROL

	Minimum	Maximum
NORMAL	68157,00	98300,00
ETANOL	62157,00	106729,00
10 nM E	88443,00	108157,00
Total	62157,00	108157,00

ANOVA

MCF7PROL

	Sum of Squares	df	Mean Square	F	Sig.
Between Groups	1030723064	2	515361531,8	4,321	,029
Within Groups	2146792246	18	119266235,9		
Total	3177515310	20			

Post Hoc Tests

Multiple Comparisons

Dependent Variable: MCF7PROL

Tukey HSD

(I) GRUP	(J) GRUP	Mean Difference (I-J)	Std. Error	Sig.	95% Confidence Interval	
					Lower Bound	Upper Bound
NORMAL	ETANOL	1262,00000	5837,47098	,975	-13636,1787	16160,1787
	10 nM E	-14190,42857	5837,47098	,063	-29088,6073	707,7501
ETANOL	NORMAL	-1262,00000	5837,47098	,975	-16160,1787	13636,1787
	10 nM E	-15452,4286*	5837,47098	,041	-30350,6073	-554,2499
10 nM E	NORMAL	14190,42857	5837,47098	,063	-707,7501	29088,6073
	ETANOL	15452,42857*	5837,47098	,041	554,2499	30350,6073

*. The mean difference is significant at the 0.05 level.
