# Supplementary material for: Estrogen and Androgen Hormone Levels Modulate the Expression of PIWI Interacting RNA in Prostate and Breast Cancer
Source: PLoS One. 2016 Jul 14;11(7):e0159044. doi: 10.1371/journal.pone.0159044 (PMC4944994; doi:10.1371/journal.pone.0159044)
Supplement: S4 File — (PDF) [file pone.0159044.s004.pdf]

GRUP

Tests of Normality

	GRUP	Kolmogorov-Smirnov[a] Statistic	df	Sig.	Shapiro-Wilk Statistic	df	Sig.
MDA_ADEZYON	1,00	,253	7	,194	,930	7	,554
	2,00	,148	7	,200*	,949	7	,716
	3,00	,142	7	,200*	,976	7	,935
MDA_RPO	1,00	,357	7	,007	,739	7	,010
	2,00	,185	7	,200*	,914	7	,427
	3,00	,244	7	,200*	,854	7	,134

*. This is a lower bound of the true significance.

a. Lilliefors Significance Correction

Oneway

Descriptives

MDA_ADEZYON

	N	Mean	Std. Deviation	Std. Error	95% Confidence Interval for Mean Lower Bound	Upper Bound
1,00	7	,7384	,05617	,02123	,6865	,7904
2,00	7	,7020	,03102	,01172	,6733	,7307
3,00	7	,6947	,04913	,01857	,6493	,7402
Total	21	,7117	,04841	,01056	,6897	,7337

MDA_ADEZYON

	Minimum	Maximum
1,00	,65	,81
2,00	,67	,75
3,00	,61	,76
Total	,61	,81

ANOVA

MDA_ADEZYON

	Sum of Squares	df	Mean Square	F	Sig.
Between Groups	,008	2	,004	1,764	,200
Within Groups	,039	18	,002		
Total	,047	20			

Nonparametric Tests

Descriptives

MDA_RPO

	N	Mean	Std. Deviation	Std. Error	Minimum	Maximum
1,00	7	20816,7143	7496,41118	2833,37710	14525,00	37025,00
2,00	7	27525,0000	7442,83772	2813,12824	18025,00	37275,00
3,00	7	32489,2857	10916,75754	4126,14651	19525,00	44275,00
Total	21	26943,6667	9656,66958	2107,25806	14525,00	44275,00

Hypothesis Test Summary

	Null Hypothesis	Test	Sig.	Decision
1	The distribution of MDA_RPO is the same across categories of GRUP.	Independent-Samples Kruskal-Wallis Test	,048	Reject the null hypothesis.

Asymptotic significances are displayed. The significance level is ,05.

Pairwise Comparisons of GRUP

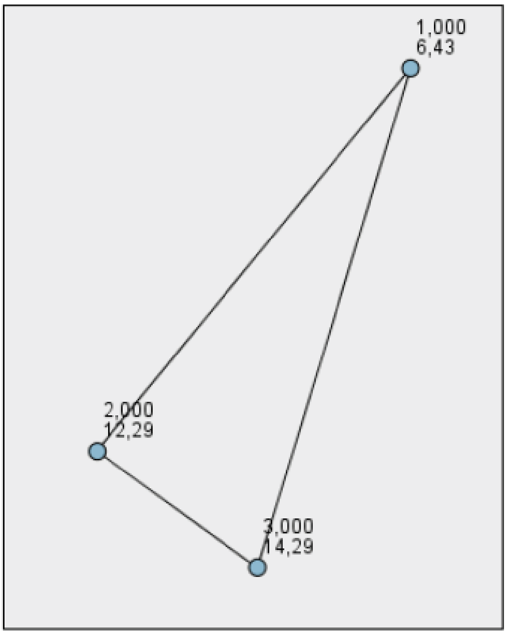

Each node shows the sample average rank of GRUP.

Sample1-Sample2	Test Statistic	Std. Error	Std. Test Statistic	Sig.	Adj.Sig.
**1,000-2,000**	-5,857	3,314	-1,767	,077	,232
**1,000-3,000**	-7,857	3,314	-2,371	,018	,053
**2,000-3,000**	-2,000	3,314	-,603	,546	1,000

Each row tests the null hypothesis that the Sample 1 and Sample 2 distributions are the same.
Asymptotic significances (2-sided tests) are displayed. The significance level [illegible]
