# Supplementary material for: Estrogen and Androgen Hormone Levels Modulate the Expression of PIWI Interacting RNA in Prostate and Breast Cancer
Source: PLoS One. 2016 Jul 14;11(7):e0159044. doi: 10.1371/journal.pone.0159044 (PMC4944994; doi:10.1371/journal.pone.0159044)
Supplement: S5 File — (PDF) [file pone.0159044.s005.pdf]

Explore

grup

Tests of Normality

	grup	Kolmogorov-Smirnov[a]			Shapiro-Wilk		
		Statistic	df	Sig.	Statistic	df	Sig.
yüzdecanlılık	NORMAL	,215	8	,200*	,925	8	,468
	ETANOL	,207	8	,200*	,918	8	,412
	1 nM ANDROJEN	,208	8	,200*	,934	8	,555

*. This is a lower bound of the true significance.

a. Lilliefors Significance Correction

Oneway

Descriptives

yüzdecanlılık

	N	Mean	Std. Deviation	Std. Error	95% Confidence ...
					Lower Bound
NORMAL	8	80,8750	9,92022	3,50733	72,5815
ETANOL	8	68,0000	9,21179	3,25686	60,2987
1 nM ANDROJEN	8	86,7500	6,84001	2,41831	81,0316
Total	24	78,5417	11,57576	2,36289	73,6537

Descriptives

yüzdecanlılık

	95% Confidence ...	Minimum	Maximum
	Upper Bound		
NORMAL	89,1685	67,00	100,00
ETANOL	75,7013	50,00	80,00
1 nM ANDROJEN	92,4684	78,00	100,00
Total	83,4297	50,00	100,00

ANOVA

yüzdecanlılık

	Sum of Squares	df	Mean Square	F	Sig.
Between Groups	1471,583	2	735,792	9,595	,001
Within Groups	1610,375	21	76,685		
Total	3081,958	23			

Post Hoc Tests

Multiple Comparisons

Dependent Variable: yüzdecanlılık

Tukey HSD

(I) grup	(J) grup	Mean Difference (I-J)	Std. Error	Sig.	95% ... Lower Bound
NORMAL	ETANOL	12,87500*	4,37849	,020	1,8387
	1 nM ANDROJEN	-5,87500	4,37849	,389	-16,9113
ETANOL	NORMAL	-12,87500*	4,37849	,020	-23,9113
	1 nM ANDROJEN	-18,75000*	4,37849	,001	-29,7863
1 nM ANDROJEN	NORMAL	5,87500	4,37849	,389	-5,1613
	ETANOL	18,75000*	4,37849	,001	7,7137

Multiple Comparisons

Dependent Variable: yüzdecanlılık

Tukey HSD

(I) grup	(J) grup	95% ... Upper Bound
NORMAL	ETANOL	23,9113
	1 nM ANDROJEN	5,1613
ETANOL	NORMAL	-1,8387
	1 nM ANDROJEN	-7,7137
1 nM ANDROJEN	NORMAL	16,9113
	ETANOL	29,7863

*. The mean difference is significant at the 0.05 level.
