# Supplementary material for: Estrogen and Androgen Hormone Levels Modulate the Expression of PIWI Interacting RNA in Prostate and Breast Cancer
Source: PLoS One. 2016 Jul 14;11(7):e0159044. doi: 10.1371/journal.pone.0159044 (PMC4944994; doi:10.1371/journal.pone.0159044)
Supplement: S6 File — (PDF) [file pone.0159044.s006.pdf]

Explore

GRUP

Tests of Normality

	GRUP	Kolmogorov-Smirnov[a] Statistic	df	Sig.	Shapiro-Wilk Statistic	df
YÜZDECANLILIK	NORMAL	,193	8	,200*	,901	8
	ETANOL	,153	8	,200*	,963	8
	10 nM ÖSTROJEN	,167	8	,200*	,912	8

Tests of Normality

	GRUP	Shapiro-... Sig.
YÜZDECANLILIK	NORMAL	,295
	ETANOL	,835
	10 nM ÖSTROJEN	,372

*. This is a lower bound of the true significance.

a. Lilliefors Significance Correction

Oneway

Descriptives

YÜZDECANLILIK

	N	Mean	Std. Deviation	Std. Error	95% Confidence ... Lower Bound
NORMAL	8	82,1250	12,41471	4,38926	71,7460
ETANOL	8	71,8750	8,27108	2,92427	64,9602
10 nM ÖSTROJEN	8	86,7500	9,54314	3,37401	78,7717
Total	24	80,2500	11,65165	2,37838	75,3299

Descriptives

YÜZDECANLILIK

	95% Confidence ... Upper Bound	Minimum	Maximum
NORMAL	92,5040	67,00	100,00
ETANOL	78,7898	57,00	83,00
10 nM ÖSTROJEN	94,7283	75,00	100,00
Total	85,1701	57,00	100,00

ANOVA

YÜZDECANLILIK

	Sum of Squares	df	Mean Square	F	Sig.
Between Groups	927,250	2	463,625	4,435	,025
Within Groups	2195,250	21	104,536		
Total	3122,500	23			

Post Hoc Tests

Multiple Comparisons

Dependent Variable: YÜZDECANLILIK

Tukey HSD

(I) GRUP	(J) GRUP	Mean Difference (I-J)	Std. Error	Sig.	95% ... Lower Bound
NORMAL	ETANOL	10,25000	5,11214	,136	-2,6355
	10 nM ÖSTROJEN	-4,62500	5,11214	,643	-17,5105
ETANOL	NORMAL	-10,25000	5,11214	,136	-23,1355
	10 nM ÖSTROJEN	-14,87500*	5,11214	,022	-27,7605
10 nM ÖSTROJEN	NORMAL	4,62500	5,11214	,643	-8,2605
	ETANOL	14,87500*	5,11214	,022	1,9895

Multiple Comparisons

Dependent Variable: YÜZDECANLILIK

Tukey HSD

(I) GRUP	(J) GRUP	95% ... Upper Bound
NORMAL	ETANOL	23,1355
	10 nM ÖSTROJEN	8,2605
ETANOL	NORMAL	2,6355
	10 nM ÖSTROJEN	-1,9895
10 nM ÖSTROJEN	NORMAL	17,5105
	ETANOL	27,7605

*. The mean difference is significant at the 0.05 level.
