# Supplementary material for: Estrogen and Androgen Hormone Levels Modulate the Expression of PIWI Interacting RNA in Prostate and Breast Cancer
Source: PLoS One. 2016 Jul 14;11(7):e0159044. doi: 10.1371/journal.pone.0159044 (PMC4944994; doi:10.1371/journal.pone.0159044)
Supplement: S7 File — (PDF) [file pone.0159044.s007.pdf]

Explore

Grup

Tests of Normality

	Grup	Kolmogorov-Smirnov[a]			Shapiro-Wilk		
		Statistic	df	Sig.	Statistic	df	Sig.
MDA_Canllk	Kontrol	,321	8	,015	,756	8	,010
	Etanol	,232	8	,200*	,861	8	,122
	1 nM ÖSTROJEN	,316	8	,018	,776	8	,016

*. This is a lower bound of the true significance.

a. Lilliefors Significance Correction

Descriptives

MDA_Canllk

	N	Mean	Std. Deviation	Std. Error	95% Confidence Interval for Mean		Minimum	Maximum
					Lower Bound	Upper Bound		
Kontrol	8	72,8750	29,52692	10,43934	48,1899	97,5601	33,00	100,00
Etanol	8	77,0000	21,58041	7,62983	58,9583	95,0417	40,00	100,00
1 nM ÖSTROJEN	8	86,1250	15,41277	5,44924	73,2396	99,0104	67,00	100,00
Total	24	78,6667	22,61332	4,61592	69,1179	88,2154	33,00	100,00

Nonparametric Tests

Hypothesis Test Summary

	Null Hypothesis	Test	Sig.	Decision
1	The distribution of MDA_Canllk is the same across categories of Grup.	Independent-Samples Kruskal-Wallis Test	,553	Retain the null hypothesis.

Asymptotic significances are displayed. The significance level is ,05.
