# Supplementary material for: Estrogen and Androgen Hormone Levels Modulate the Expression of PIWI Interacting RNA in Prostate and Breast Cancer
Source: PLoS One. 2016 Jul 14;11(7):e0159044. doi: 10.1371/journal.pone.0159044 (PMC4944994; doi:10.1371/journal.pone.0159044)
Supplement: S8 File — (PDF) [file pone.0159044.s008.pdf]

Explore

grup

Tests of Normality

	grup	Kolmogorov-Smirnov[a] Statistic	df	Sig.	Shapiro-Wilk Statistic	df	Sig.
adezyon	NORMAL	,234	7	,200*	,856	7	,139
	ETANOL	,285	7	,089	,871	7	,190
	1 nM DHT	,356	7	,008	,719	7	,006

*. This is a lower bound of the true significance.

a. Lilliefors Significance Correction

Percentiles

		grup	Percentiles 25	50
Weighted Average(Definition 1)	adezyon	NORMAL	,8630	,8920
		ETANOL	,8870	,9110
		1 nM DHT	,7870	,8000

Percentiles

		grup	Percentiles 75
Weighted Average(Definition 1)	adezyon	NORMAL	,9710
		ETANOL	,9280
		1 nM DHT	,8680

Descriptives

		N	Mean	Std. Deviation	Std. Error	95% Confidence ... Lower Bound
adezyon	NORMAL	7	,9256	,09285	,03510	,8397
	ETANOL	7	,8997	,07023	,02655	,8348
	1 nM DHT	7	,7874	,11586	,04379	,6803
	Total	21	,8709	,10894	,02377	,8213

Descriptives

		95% Confidence ... Upper Bound	Minimum	Maximum
adezyon	NORMAL	1,0114	,84	1,11
	ETANOL	,9647	,76	,99
	1 nM DHT	,8946	,54	,87
	Total	,9205	,54	1,11

Nonparametric Tests

Hypothesis Test Summary

	Null Hypothesis	Test	Sig.	Decision
1	The distribution of adezyon is the same across categories of grup.	Independent-Samples Kruskal-Wallis Test	,018	Reject the null hypothesis.

Asymptotic significances are displayed. The significance level is ,05.

Pairwise Comparisons of grup

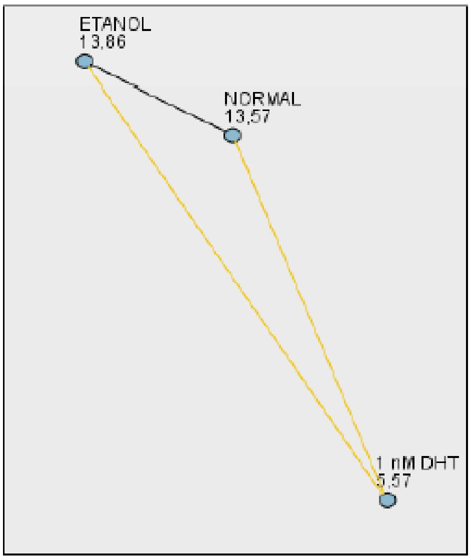

Each node shows the sample average rank of grup.

Sample1-Sample2	Test Statistic	Std. Error	Std. Test Statistic	Sig.	Adj.Sig.
1 nM DHT-NORMAL	8,000	3,317	2,412	,016	,048
1 nM DHT-ETANOL	8,286	3,317	2,498	,012	,037
NORMAL-ETANOL	-,286	3,317	-,086	,931	1,000

Each row tests the null hypothesis that the Sample 1 and Sample 2 distributions are the same.
Asymptotic significances (2-sided tests) are displayed. The significance level
