# Supplementary material for: Estrogen and Androgen Hormone Levels Modulate the Expression of PIWI Interacting RNA in Prostate and Breast Cancer
Source: PLoS One. 2016 Jul 14;11(7):e0159044. doi: 10.1371/journal.pone.0159044 (PMC4944994; doi:10.1371/journal.pone.0159044)
Supplement: S9 File — (PDF) [file pone.0159044.s009.pdf]

Explore

grup

Tests of Normality

	grup	Kolmogorov-Smirnov[a]			Shapiro-Wilk		
		Statistic	df	Sig.	Statistic	df	Sig.
pir1651	MCF7 KONTROL	,108	7	,200*	,978	7	,949
	MCF7 Östrojen	,108	7	,200*	,978	7	,949
	LNCAP KONTROL	,108	7	,200*	,978	7	,949
	LNCAP ANDROJEN	,108	7	,200*	,978	7	,949
	MDA KONTROL	,108	7	,200*	,978	7	,949
	MDA ÖSTROJEN	,108	7	,200*	,978	7	,949
pir823	MCF7 KONTROL	,108	7	,200*	,978	7	,949
	MCF7 Östrojen	,108	7	,200*	,978	7	,949
	LNCAP KONTROL	,108	7	,200*	,978	7	,949
	LNCAP ANDROJEN	,108	7	,200*	,978	7	,949
	MDA KONTROL	,108	7	,200*	,978	7	,949
	MDA ÖSTROJEN	,108	7	,200*	,978	7	,949

*. This is a lower bound of the true significance.

a. Lilliefors Significance Correction

T-Test

Group Statistics

	grup	N	Mean	Std. Deviation	Std. Error Mean
pir1651	MCF7 KONTROL	7	,500000000	,0002160247	,0000816497
	MCF7 Östrojen	7	,697400000	,0002160247	,0000816497
pir823	MCF7 KONTROL	7	,539600000	,0002160247	,0000816497
	MCF7 Östrojen	7	,271700000	,0002160247	,0000816497

Independent Samples Test

		Levene's Test for Equality of Variances		t-test for Equality of Means	
		F	Sig.	t	df
pir1651	Equal variances assumed	,000	1,000	-1709,534	12
	Equal variances not assumed			-1709,534	12,000
pir823	Equal variances assumed	,000	1,000	2320,082	12
	Equal variances not assumed			2320,082	12,000

Independent Samples Test

		t-test for Equality of ...
		Sig. (2-tailed)
pir1651	Equal variances assumed	,000
	Equal variances not assumed	,000
pir823	Equal variances assumed	,000
	Equal variances not assumed	,000

T-Test

Group Statistics

	grup	N	Mean	Std. Deviation	Std. Error Mean
pir1651	LNCAP KONTROL	7	,003200000	,0002160247	,0000816497
	LNCAP ANDROJEN	7	,014900000	,0002160247	,0000816497
pir823	LNCAP KONTROL	7	,004800000	,0002160247	,0000816497
	LNCAP ANDROJEN	7	,017800000	,0002160247	,0000816497

Independent Samples Test

		Levene's Test for Equality of Variances		t-test for Equality of Means	
		F	Sig.	t	df
pir1651	Equal variances assumed	,000	1,000	-101,325	12
	Equal variances not assumed			-101,325	12,000
pir823	Equal variances assumed	,000	1,000	-112,583	12
	Equal variances not assumed			-112,583	12,000

Independent Samples Test

		t-test for Equality of ...
		Sig. (2-tailed)
pir1651	Equal variances assumed	,000
	Equal variances not assumed	,000
pir823	Equal variances assumed	,000
	Equal variances not assumed	,000

T-Test

Group Statistics

	grup	N	Mean	Std. Deviation	Std. Error Mean
pir1651	MDA KONTROL	7	,607100000	,0002160247	,0000816497
	MDA ÖSTROJEN	7	12,46660000	,0002160247	,0000816497
pir823	MDA KONTROL	7	2,042000000	,0002160247	,0000816497
	MDA ÖSTROJEN	7	9,513700000	,0002160247	,0000816497

Independent Samples Test

		Levene's Test for Equality of Variances		t-test for Equality of Means	
		F	Sig.	t	df
pir1651	Equal variances assumed	,000	1,000	-102706,283	12
	Equal variances not assumed			-102706,283	12,000
pir823	Equal variances assumed	,000	1,000	-64706,820	12
	Equal variances not assumed			-64706,820	12,000

Independent Samples Test

		t-test for Equality of ...
		Sig. (2-tailed)
pir1651	Equal variances assumed	,000
	Equal variances not assumed	,000
pir823	Equal variances assumed	,000
	Equal variances not assumed	,000
