# Supplementary material for: Estrogen and Androgen Hormone Levels Modulate the Expression of PIWI Interacting RNA in Prostate and Breast Cancer
Source: PLoS One. 2016 Jul 14;11(7):e0159044. doi: 10.1371/journal.pone.0159044 (PMC4944994; doi:10.1371/journal.pone.0159044)
Supplement: S10 File — (PDF) [file pone.0159044.s010.pdf]

Explore

grup

Tests of Normality

	grup	Kolmogorov-Smirnov[a]			Shapiro-Wilk		
		Statistic	df	Sig.	Statistic	df	Sig.
pir_651	Kontrol	,132	7	,200[*]	,965	7	,863
	Andojen 10 nM	,108	7	,200[*]	,978	7	,949
pir_823	Kontrol	,108	7	,200[*]	,978	7	,949
	Andojen 10 nM	,108	7	,200[*]	,978	7	,949

*. This is a lower bound of the true significance.

a. Lilliefors Significance Correction

T-Test

Group Statistics

	grup	N	Mean	Std. Deviation	Std. Error Mean
pir_651	Kontrol	7	,243200	,0002944	,0001113
	Andojen 10 nM	7	1,765400	,0002160	,0000816
pir_823	Kontrol	7	,558600	,0002160	,0000816
	Andojen 10 nM	7	1,239700	,0002160	,0000816

Independent Samples Test

		Levene's Test for Equality of Variances		t-test for Equality of Means	
		F	Sig.	t	df
pir_651	Equal variances assumed	,600	,454	-11029,387	12
	Equal variances not assumed			-11029,387	11,009
pir_823	Equal variances assumed	,000	1,000	-5898,499	12
	Equal variances not assumed			-5898,499	12,000

Independent Samples Test

		t-test for Equality of ...
		Sig. (2-tailed)
pir_651	Equal variances assumed	,000
	Equal variances not assumed	,000
pir_823	Equal variances assumed	,000
	Equal variances not assumed	,000

Explore

grup

Tests of Normality

		Kolmogorov-Smirnov[a]			Shapiro-Wilk		
	grup	Statistic	df	Sig.	Statistic	df	Sig.
pir_651	Kontrol	,132	7	,200*	,965	7	,863
	Andojen 10 nM	,108	7	,200*	,978	7	,949
	Etanol	,108	7	,200*	,978	7	,949
	Andojen 10 nM	,108	7	,200*	,978	7	,949
pir_823	Kontrol	,108	7	,200*	,978	7	,949
	Andojen 10 nM	,108	7	,200*	,978	7	,949
	Etanol	,108	7	,200*	,978	7	,949
	Andojen 10 nM	,108	7	,200*	,978	7	,949

*. This is a lower bound of the true significance.

a. Lilliefors Significance Correction

T-Test

Notes

Output Created		29-MAY-2015 14:04:13
Comments		
Input	Data	C:\Users\pc\Desktop\PC3 gen ekspresyonu.sav
	Active Dataset	DataSet0
	Filter	<none>
	Weight	<none>
	Split File	<none>
	N of Rows in Working Data File	28
Missing Value Handling	Definition of Missing	User defined missing values are treated as missing.
	Cases Used	Statistics for each analysis are based on the cases with no missing or out-of-range data for any variable in the analysis.
Syntax		T-TEST GROUPS=grup(3 4)<br>/MISSING=ANALYSIS<br>/VARIABLES=pir_651 pir_823<br>/CRITERIA=CI(.95).
Resources	Processor Time	00:00:00,02
	Elapsed Time	00:00:00,02

Group Statistics

	grup	N	Mean	Std. Deviation	Std. Error Mean
pir_651	Etanol	7	4,112000	,0021602	,0008165
	Andojen 10 nM	7	6,589000	,0021602	,0008165
pir_823	Etanol	7	1,790000	,0021602	,0008165
	Andojen 10 nM	7	2,014000	,0021602	,0008165

Independent Samples Test

		Levene's Test for Equality of Variances		t-test for Equality of Means	
		F	Sig.	t	df
pir_651	Equal variances assumed	,000	1,000	-2145,145	12
	Equal variances not assumed			-2145,145	12,000
pir_823	Equal variances assumed	,000	1,000	-193,990	12
	Equal variances not assumed			-193,990	12,000

Independent Samples Test

		t-test for Equality of ...
		Sig. (2-tailed)
pir_651	Equal variances assumed	,000
	Equal variances not assumed	,000
pir_823	Equal variances assumed	,000
	Equal variances not assumed	,000
